# Association between hypertension and hearing loss: a systemic review and meta-analysis

**DOI:** 10.3389/fneur.2024.1470997

**Published:** 2025-01-07

**Authors:** Xiaohua Jin, Xianpeng Xu, Jingjing Wang, Xinghong Liu, Xinxing Deng, Hui Xie

**Affiliations:** ^1^Department of Otorhinolaryngology, Chengdu University of Traditional Chinese Medicine, Chengdu, China; ^2^Department of Otorhinolaryngology, Hospital of Chengdu University of Traditional Chinese Medicine, Chengdu, China

**Keywords:** hearing loss, hypertension, systolic pressure, diastolic pressure, meta-analysis

## Abstract

**Objective:**

To systematically evaluate the association between hypertension and hearing loss.

**Methods:**

A standardized search for studies on hypertension and hearing loss in PubMed, Embase, Scopus, and Web of Science was performed using subject terms, free terms, and keyword combinations for the period of library construction to March 2024. Meta-analysis was performed using RevMan 5.4 and STATA 18.0.

**Results:**

A total of 12 studies were included, assessing 594,676 participants. The combined OR using the random effects model was 1.849 (95% CI: 1.549, 2.208). Heterogeneity in this analysis was high (*I*^2^ = 98%, *p* < 0.1), and by sensitivity analysis we found that the heterogeneity may have originated from 3 studies, the removal of which significantly reduced the heterogeneity and had a small effect on the effect size [OR (95%CI): 1.893 (1.834, 1.953), *I*^2^ = 0.0%, *p* = 0.465].

**Conclusion:**

Hypertension may be one of the risk factors for hearing loss. Identification of hypertension can help in early assessment and management of hearing loss risk.

**Systematic review registration:**

https://www.crd.york.ac.uk/prospero/, identifier CRD42023460001.

## Introduction

As an escalating concern in the realm of public health, hearing impairment has garnered global focus. As per a definitive report from the World Health Organization (WHO), approximately 466 million individuals across the globe grapple with hearing loss (1). Moreover, in 2015, the global expenditure for addressing hearing loss surged to an estimated $67 to $107 billion (2). The ramifications of hearing impairment extend far beyond mere statistics. It not only imposes a substantial economic strain on the global healthcare infrastructure, but also diminishes patients’ capacity for communication, resulting in a decline in social engagement and an upsurge in the incidence of depression, cognition, dementia, and other related ailments (3–5).

It is now widely recognized that aging, use of ototoxic medications, noise exposure lead to hearing loss (6, 7). However, the link between hypertension and hearing loss is still controversial. Havilah’s (8) cross-sectional study of 137 participants revealed a notable link between hypertension and age-related hearing impairment. Similarly, Agarwa’s (9) case–control study affirmed a significant association between the two. Conversely, Miyata’s (10) retrospective longitudinal study indicated a correlation between higher systolic blood pressure at 1 kHz and hearing loss, with no evident connection at 4 kHz. However, Shargorodsky’s (11) prospective cohort study arrived at divergent conclusions, finding no substantial correlation between hypertension and an elevated risk of hearing impairment. In addition, Engdahl’s (12) cohort study based on 31,547 subjects shows that there is a correlation between hypertension and hearing loss, but it is pointed out that this effect is very small and its clinical relevance was questionable.

These divergent outcomes imply that the interplay between hypertension and hearing loss might be swayed by an array of factors, including sample size, study methodology, and subject traits. Hence, it becomes paramount to clarify and elucidate and quantify the connection between hypertension and hearing impairment.

The aim of this study was to conduct a comprehensive review of pertinent literature, aiming to evaluate and quantify the correlation between hypertension and hearing loss. Furthermore, we sought to pinpoint potential sources of variation among studies through meta-regression analysis.

## Materials and methods

### Search strategy

Our investigation revolved around the PICO criteria (13), focusing on the research question “the relationship between hypertension and hearing loss.” We conducted a thorough search for eligible research across various databases, including PubMed, Embase, Scopus, and Web of Science. The retrieval strategy involved subject words and keywords, without language or time constraints. Hearing loss was defined as an average hearing threshold of ≥20 dB, while hypertension was defined as systolic blood pressure (SBP) ≥ 140 mmHg, diastolic blood pressure (DBP) ≥ 90 mmHg, or the reported use of antihypertensive drugs (14, 15). Studies were identified through a combination of terms such as sudden deafness, hearing loss, hypoacusis, hearing impairment*, deafness*, along with terms related to hypertension, high blood pressure*, hypertensive. This approach aimed to ensure that the studies might offer pertinent information on the association between hearing loss and hypertension. Additionally, we meticulously examined the reference lists of all identified studies and reviewed the cited literature to guarantee that relevant studies were not overlooked. This study has been registered as CRD 42023460001 in the International Prospective Systematic Review Registry (PROSPERO).

### Inclusion and exclusion criteria

The inclusion criteria were delineated as follows: Study population encompassed all ages and genders without restriction. Study types included case–control studies, cross-sectional studies, and cohort studies. The exposure factor under scrutiny was hearing loss, while the outcome of interest was hypertension. Additionally, the study data needed to be comprehensive, allowing for the derivation of OR and 95% confidence intervals, or the provision of relevant data to facilitate their calculation.

Exclusion criteria were as follows: unclear diagnostic criteria; incomplete data; study type: qualitative, policy, opinion, case-reports, review, case studies.

### Study selection and data extraction

The search was independently conducted by two authors (Jin and Xu), who screened the titles and abstracts of all literature. Those that met the inclusion criteria were thoroughly reviewed, and pertinent data were extracted. In cases of discrepancies, the decision to include literature and resolve differences in extracted data was reached through mutual discussion between the two authors. In the event of discord, a resolution would be achieved through consultation with a third author. The extracted data encompassed authors’ names, year of publication, country, study type, percentage of female participants, methods for determining hearing loss and hypertension, the number of hypertensive individuals within the hearing-impaired population, and the number within the normal hearing population. For this study, the OR was selected as the measure of effect index, providing a partial reflection of the strength of the association between hypertension and hearing loss. In instances where multiple estimates were reported within the same study, priority was given to the pooled analysis utilizing the model with the most comprehensive adjustments to ensure the accuracy and reliability of the analytical results (16).

### Quality assessment

The Newcastle-Ottawa Scale (NOS) was employed to assess the research quality of case–control studies, yielding scores ranging from 0 to 9 stars. Documents scoring ≤5 were categorized as low quality, while those scoring ≥6 were deemed high quality (Annexure 2a) (17, 18). This cross-sectional study utilized the Agency for Healthcare Research and Quality (AHRQ) to appraise the quality of the 11-level research, defining scores of 0–3, 4–7, and 8–11 as low, medium, and high quality (Annexure 2b) (17, 18). The quality assessment was independently conducted by two authors, with any discrepancies resolved through mutual consultation.

### Data analysis

The data underwent analysis using Review Manager 5.4 and STATA 18.0, with the OR serving as the effect indicator. The strength of the association between hypertension and hearing loss was ascertained by amalgamating the adjusted ORs and their respective 95% confidence intervals. Heterogeneity within the studies was gauged using the I^2^ statistic and Cochran’s q test. In instances of high heterogeneity (*I*^2^ ≥ 50%, *p* ≤ 0.1), the random effect model was employed, while in cases of low heterogeneity (*I*^2^ ≤ 50%, *p* > 0.1), the fixed effect model was utilized (19). For high heterogeneity, sensitivity analysis was employed to discern potential sources of variation on a case-by-case basis (20). Subgroup analyses were conducted based on study area, type of study, year of publication, sample size, age of participants, percentage of females, mode of hearing diagnosis, and whether or not relevant con founders (occupational exposures, diabetes mellitus, high blood cholesterol, and ototoxic medication use) were excluded from the inclusion of study participants, and meta-regression analyses were conducted to further identify sources of heterogeneity by using the above study factors as covariates (21). Publication bias was assessed via funnel plot, Egger, and Begg correlation tests (22–24). A *p*-value below 0.05 is considered to have statistical significance.

### Language proofreading

For the language proofreading of the manuscript, we used ChatGPT-3.5 of GPT.

## Results

### Search steps and screening results

**Figure 1:**
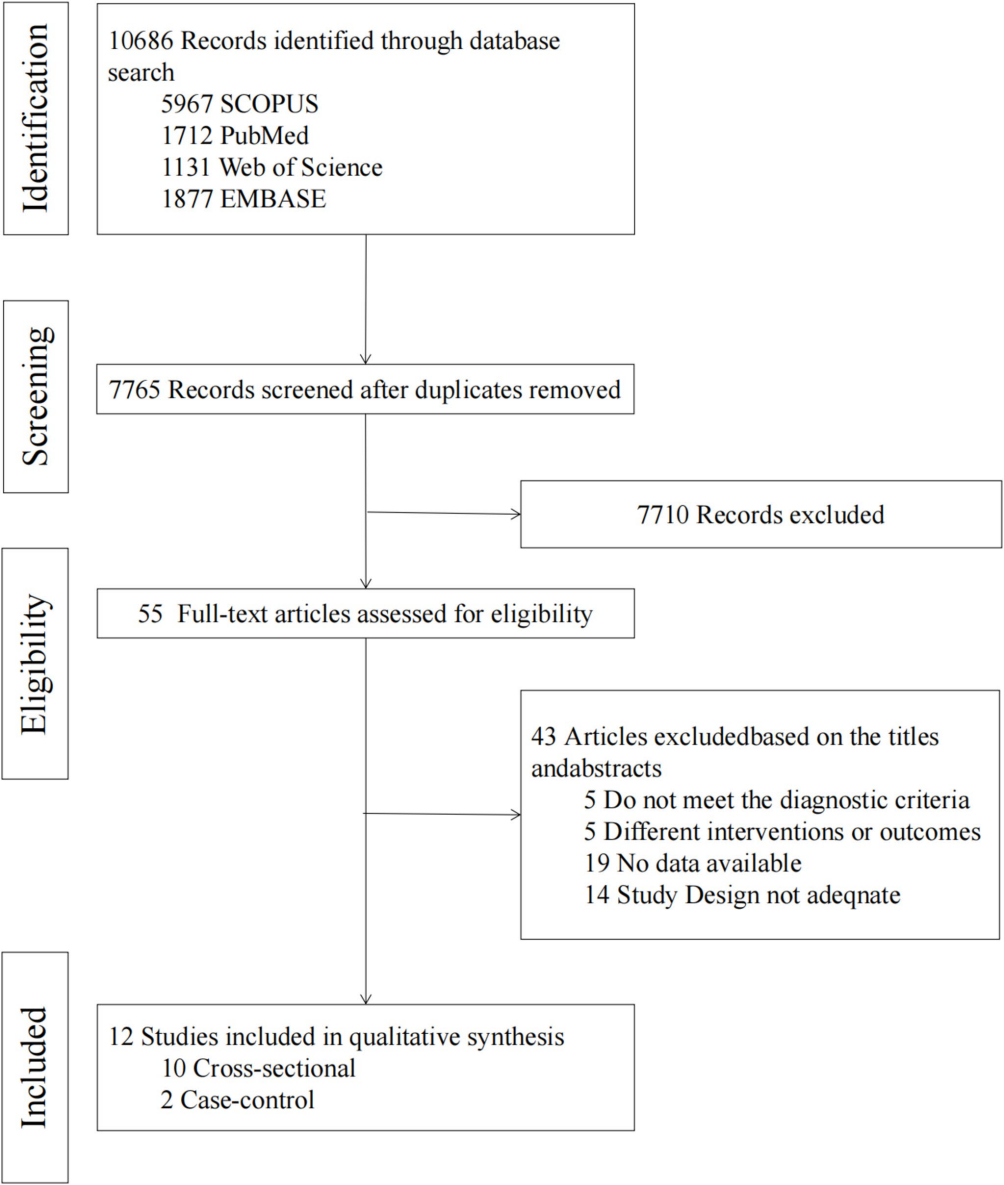
Literature screening process and results.

A total of 10,686 documents were obtained through the systematic search, and after removing duplicates, 7,765 documents were screened for titles/abstracts (Annexure 3). From these, 7,710 documents were removed by title/abstract, and 55 documents were finally screened for full text. Of these, 43 were removed because they did not meet the inclusion criteria (Annexure 4). Finally, a total of 12 articles were included in the meta-analysis. The flowchart depicts the article retrieval and screening process (Figure 1).

### Basic characteristics

This study included 12 studies with a total of 594,676 participants, and its basic features are shown in Tables 1, 2. The study spans from 2006 to 2023 and comprises cross-sectional studies (10 items) and case–control studies (2 items). Eight studies were conducted in Asia, two in South America, one in Europe, and one in Africa. The average age across the studies ranged from 32.32 to 60.80 years. Five of the studies established a favorable correlation between hypertension and hearing impairment, while four studies found that the connection between hypertension and hearing loss lost significance upon adjustment for confounding factors (such as age, gender, and noise exposure). Hara’s (25) investigation revealed an independent and positive link between hypertension and hearing loss in men, with a adjusted prevalence ratio (PR) of 1.52 (95% CI: 1.07 ± 2.16), yet no such association was observed in women. Similarly, Kuang’s (26) study presented analogous outcomes, showing a correlation between hypertension and hearing loss in men, while the difference in women was not statistically significant. Umesawa’s (27) investigation revealed that the average prevalence of mild hearing loss (25 dB < PTA ≤ 40 dB) among hypertensive individuals surpasses that of non-hypertensive individuals. Nevertheless, no correlation was observed between hypertension and the average occurrence of moderate to severe hearing loss (PTA > 40 dB).

**Table 1:** Basic characteristics.

Studies	Year	Country	Study design	Age group (mean)	Women (%)	Diagnosis of depression	Diagnosis of hearing loss (dB)	N Participants	Participants with hearing loss		Participants without hearing loss		Adjusted OR (CI)
									Total	Participants with hypertension	Total	Participants with hypertension	
Zhang (27)	2023	China	CS	39.17	22.82	Clinical diagnosis	BHFTA ≥40^c^	242,811	20,497	4,409	222,314	27,792	1.07 (1.03–1.11)
Umesawa (23)	2019	Japan	CS	49.4	13.65	Clinical diagnosis	1 kHz ≥ 30 and/or 4 kHz ≥ 40	13,475	980	330	12,495	2,708	1.003 (1.001–1.005)
Hara (21)	2020	Japan	CS	60.8	63.27	Clinical diagnosis	PTA>25	1,010	250	152	760	347	NR
Ramatsoma (32)	2022	South Africa	CS	44.26	53.53	Clinical diagnosis	PTA > 20	198	53	40	145	66	4.18 (1.02–17.01)
Oh (30)	2014	Korea	CS	44.1	24.09	Clinical diagnosis	PTA ≥ 26	37,773	2,636	397	35,137	3,241	1.098 (0.93–1.83)
Guo (31)	2021	China	CS	37.06	100.00	Clinical diagnosis	PTA ≥ 26	20,882	4,024	420	16,858	1,018	NR
Samelli (28)	2021	Brazil	CS	49.00	52.78	Clinical diagnosis	PTA>25	900	439	172	461	128	NR
Wang (29)	2018	China	CS	33.64	23.56	Clinical diagnosis	PTA > 25	267,766	113,470	13,223	154,296	14,378	1.046 (1.032–1.060)
Kuang (22)	2019	China	CS	39.49	29.01	Clinical diagnosis	BHFTA >25	21,403	1,501	218	19,902	1,037	NR
Zhou (26)	2019	China	CS	32.32	20.46	Clinical diagnosis	PTA > 25	1,213	357	45	856	56	NR
Moraes (25)	2006	Brazil	CC	54.45	67.55	Clinical diagnosis	PTA > 20	308	154	72	154	46	1.73 (1.05–2.85)
Aimoni (24)	2009	Italy	CC	54.86	47.33	History of hypertension	Diagnostic criteria for sudden deafness	412	141	47	271	90	0.94 (0.58–1.53)

CS, Cross-sectional; CC, Case–control; NR, not reported. BHFTA: arithmetic mean of binaural hearing thresholds. PTA: mean pure tone hearing threshold. Diagnostic criteria for sudden deafness: a sudden hearing loss (>30 dB HL), within 3 frequencies, developing over 72 h; NR, not reported.

**Table 2:** Characterization table for exclusion of confounders in the inclusion of study participants.

Studies	Smoke	Drink wine	Occupational exposure	Diabetes	Hyperlipoidemia	Ototoxicity drug use
Zhang (27)	No	No	No	No	No	Yes
Umesawa (23)	No	No	Yes	No	No	No
Hara (21)	No	No	No	No	No	No
Ramatsoma (32)	Yes	No	Yes	No	No	Yes
Oh (30)	No	No	No	No	No	No
Guo (31)	No	No	No	Yes	Yes	Yes
Samelli (28)	No	No	No	No	No	No
Wang (29)	No	No	No	No	No	No
Kuang (22)	No	No	No	No	No	No
Zhou (26)	No	No	No	No	No	Yes
Moraes (25)	No	No	Yes	Yes	Yes	Yes
Aimoni (24)	No	No	No	No	No	Yes

### Quality evaluation of studies

NOS and AHRQ were used to evaluate the quality of case–control studies and cross-sectional studies, respectively. The two case–control studies scored 6 points and 7 points, respectively, and were rated as high quality research (28, 29). Among 10 cross-sectional studies, 4 were rated as 9 points (26, 27, 30, 31), 4 as 8 points (25, 32–34), 1 as 7 points (35), and 1 as 6 points (Annexures 2a,b) (36). In total, 10 articles were scrutinized with high quality, and 2 with medium quality.

### Heterogeneity test

**Figure 2:**
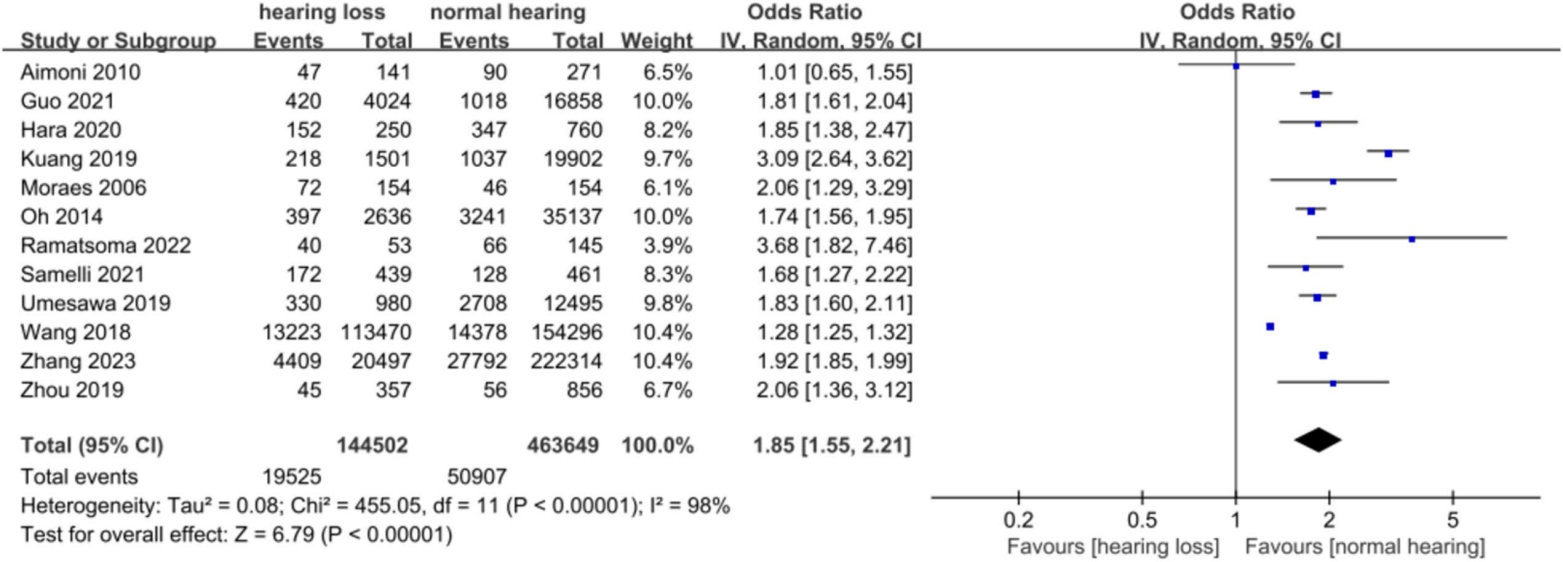
Forest diagram of the relationship between hypertension and hearing.

**Figure 3:**
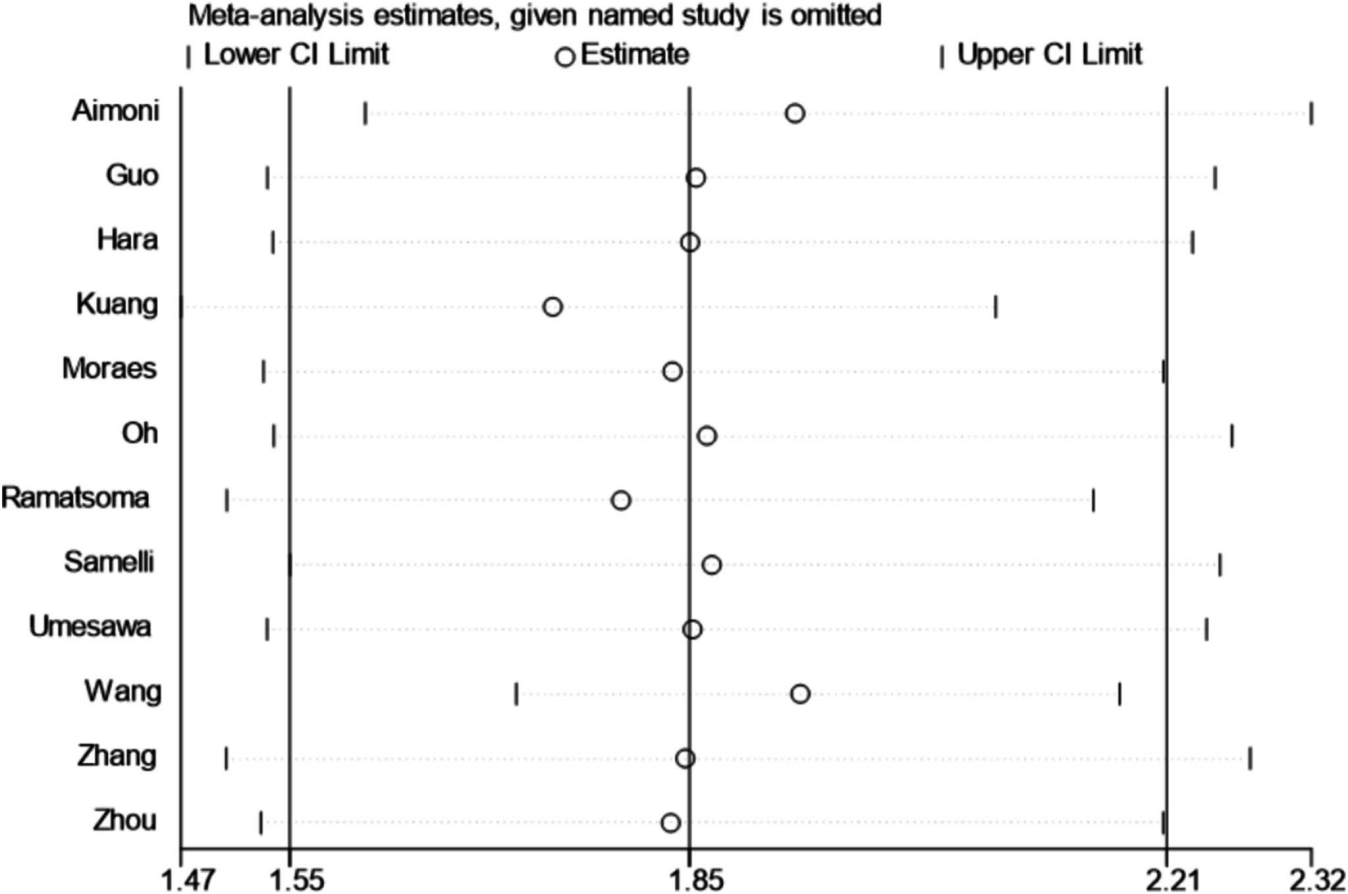
Sensitivity analysis.

A total of 12 articles were included. Following the assessment for heterogeneity, *I*^2^ = 98% > 50%, and Q test *p* < 0.00001 (Figure 2), indicating a statistically significant variance, signifying a substantial degree of heterogeneity among the included documents. In light of this circumstance, we embarked on comprehensive research and analysis, conducting a sensitivity analysis on the 12 collected articles this time (Figure 3). Sensitivity analysis was carried out by excluding individual documents one by one. We discovered that Aimoni, Kuang, and Wang significantly impacted the overall heterogeneity. To mitigate this, we opted to exclude these three studies and reevaluate the heterogeneity among the remaining nine studies. The outcomes revealed a noteworthy reduction in heterogeneity after removing the three studies (*I*^2^ = 0% < 50%, *p* = 0.46 > 0.1) (Annexure 5). Employing the fixed effect model to amalgamate the effect index [OR (95%CI): 1.89 (1.83–1.95), *p* < 0.05], as compared to the effect index of articles without the exclusion of high heterogeneity [random effect model, OR (95%CI): 1.85 (1.55–2.21), *p* < 0.05], yielded no significant variance. This outcome further corroborated the resilience of our analytical approach and findings.

### Bias risk assessment

**Figure 4:**
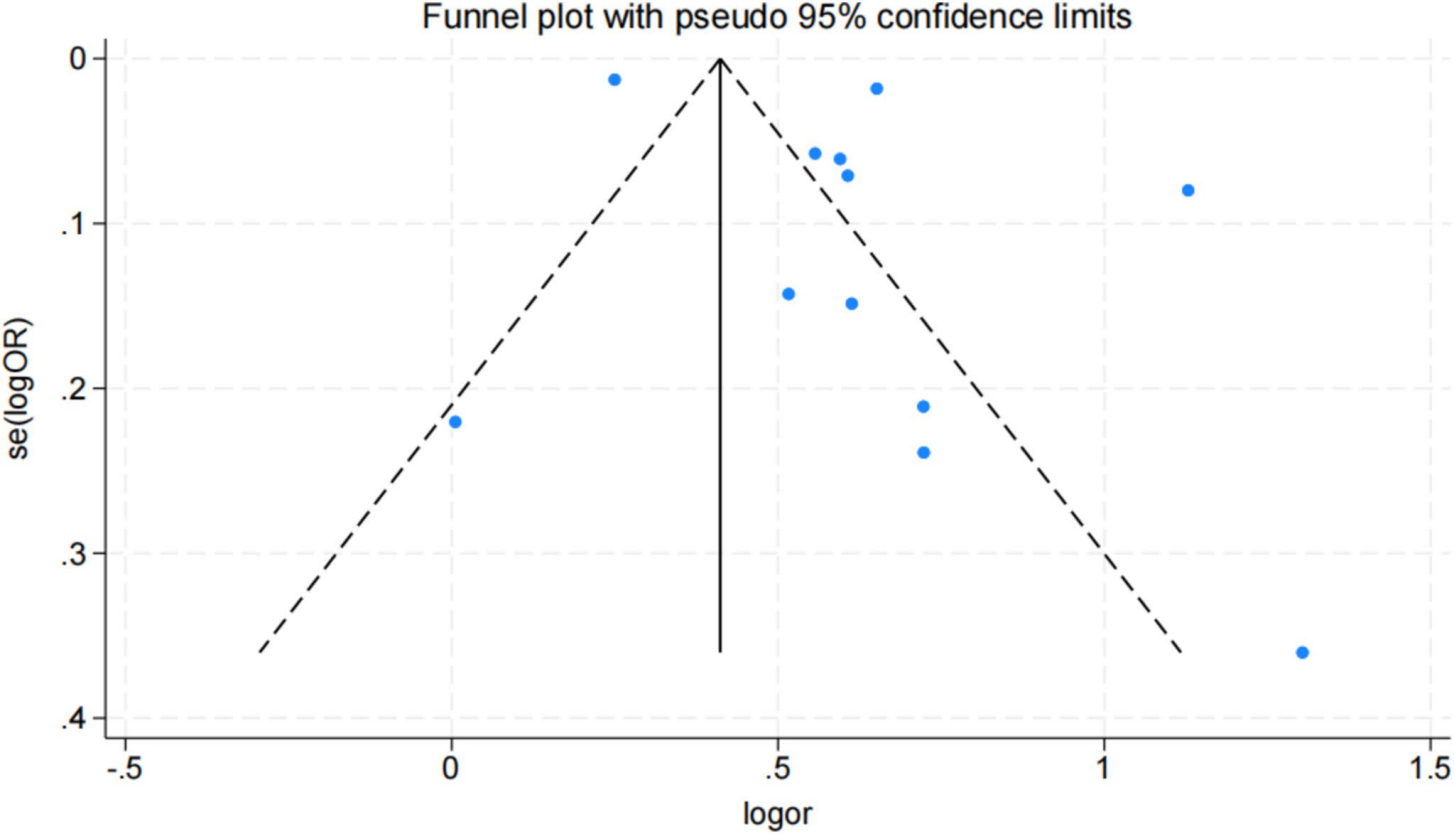
Funnel diagram.

Funnel plot, Egger, and Begg correlation tests were employed to discern the presence of publication bias. Through the funnel diagram (Figure 4), we can observe that it has asymmetry, but the funnel diagram results cannot accurately reflect whether there is publication bias or not, so we carried out further validation. The results of Begg’s bias test based on the funnel diagram showed that *p* = 0.187 > 0.05, which suggests that there is no significant publication bias in our study (Annexure 6). Additionally, the Egger test (*p* = 0.155 > 0.05) and Begg test (*p* = 0.37 > 0.05) were also conducted, further supporting the absence of publication bias in this study (Annexure 7). Summarizing the results of the above tests we concluded that the results of the current study were reliable and unaffected by publication bias.

### Mate analysis of hypertension and hearing loss

**Figure 5:**
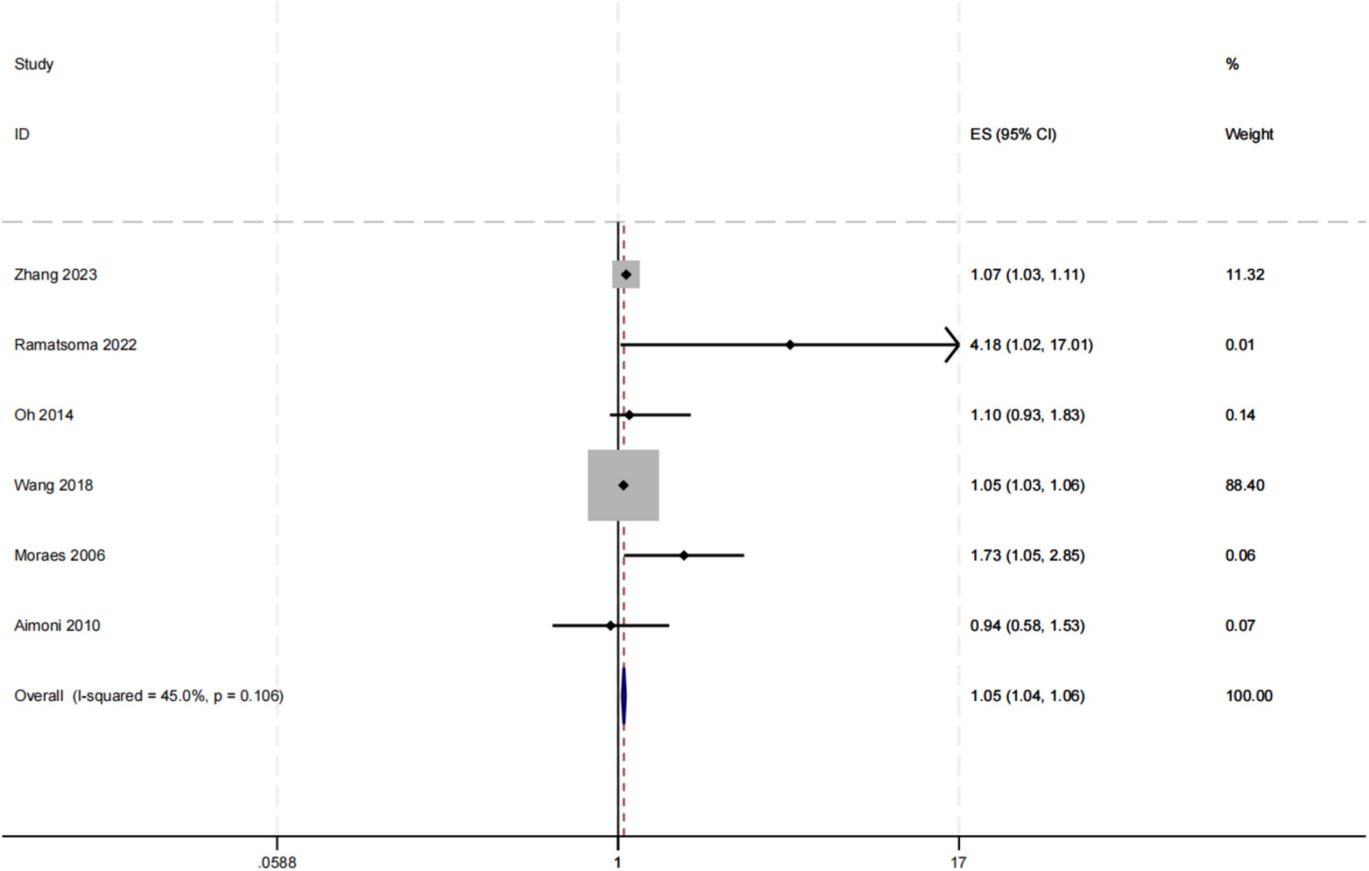
Forest map with combined adjusted effect values.

We conducted a random effect meta-analysis on the association between hypertension and hearing loss, including 10 cross-sectional studies and 2 case–control studies. After analyzing the data with the random effect model, we got the combined effect index [OR (95%CI): 1.849 (1.549, 2.208), *I*^2^ = 98%, *p* < 0.1] (Table 3). This result showed that there was a significant positive correlation between hypertension and hearing loss. Meta-analysis of the adjusted effect index also showed that there was a positive correlation between hypertension and hearing loss, but the effect index was lower than that before adjustment [OR (95%CI): 1.049 (1.036, 1.062), *I*^2^ = 45%, *p* = 0.106] (Figure 5).

**Table 3:** Subgroup analysis and meta-regression of the effect measurement.

		N studies	Subgroup analysis		
			OR (95%CI) [*p*-value]	Heterogeneity – I2; *p*-value	Meta-regression *p* value
All studies		12	1.849 (1.549, 2.208) [<0.001]	97.6%; <0.001	–
Remove high heterogeneity		9	1.893 (1.834, 1.953) [<0.001]	0.0%; 0.465	–
Adjusted OR		6	1.049 (1.036, 1.062) [<0.001]	45%; 0.106	–
Country					
Europe		1	1.006 (0.653, 1.549) [0.980]	–	0.138
Asia		8	1.884(1.534, 2.313) [<0.001]	98.4%; 0.0788	
South America		2	1.770 (1.392, 2.25) [<0.001]	0.0%; <0.0010	
Africa		1	3.683 (1.818, 7.461) [<0.001]	–	
Age					
<40 years		5	1.934 (1.470, 2.544) [<0.001]	99.1%; <0.001	0.263
40–50 years		4	1.804 (1.602, 2.032) [<0.001]	34.0%; 0.208	
50–60 years		2	1.431 (0.708, 2.892) [0.318]	79.5%; 0.027	
>60 years		1	1.846 (1.380, 2.470) [<0.001]	–	
Research type					
Case–control		2	1.431 (0.708, 2.892) [0.318]	79.5%; 0.027	0.095
Cross-sectional		10	1.920 (1.588, 2.322) [<0.001]	98.0%; <0.001	
Gender (female proportion, %)					
<50		7	1.781 (1.416, 2.241) [<0.001]	98.6%; <0.001	0.105
>50		5	1.845 (1.640, 2.075) [<0.001]	9.5%; 0.352	
Sample size					
<10,000		6	1.8059 (1.405, 2.321) [<0.001]	57.9%; 0.037	0.297
>10,000		6	1.870 (1.485, 2.355) [<0.001]	98.9%; <0.001	
Publication year					
2000–2009		1	2.062 (1.291, 3.293) [0.002]	–	0.682
2010–2019		6	1.749 (1.297, 2.358) [<0.001]	97.0%; <0.001	
2020–2023		5	1.887 (1.765, 2.019) [<0.001]	19.9%; 0.288	
Diagnostic criteria of hearing					
>20 dB		2	2.588 (1.485, 4.510) [0.001]	44.5%; 0.179	0.087
≥26 dB		6	1.683 (1.380, 2.052) [<0.001]	92.6%; <0.001	
Others		4	1.938 (1.495, 2.513) [<0.001]	93.2%; <0.001	
Exclusion of confounding factors when included in the study					
Occupational exposure	No	9	1.704 (1.417, 2.048) [<0.001]	89.4%; <0.001	0.065
	Yes	3	2.186 (1.712, 2.792) [<0.001]	94.4%; <0.001	
Diabetes/hyperlipoidemia	No	10	1.864 (1.466, 2.369) [<0.001]	95.2%; <0.001	0.152
	Yes	2	1.859 (1.703, 2.028) [<0.001]	59.4%; 0.117	
Ototoxicity drug use	No	6	1.835 (1.693, 1.989) [<0.001]	0.0%; 0.490	0.256
	Yes	6	1.771 (1.372, 2.285) [<0.001]	98.8%; <0.001	

OR, Odds Ratio.

### Subgroup analysis and meta-regression

In this investigation, a series of subgroup analyses and meta-regression analyses were employed to examine the potential relationship between hypertension and hearing loss. The results of the study showed that the association between hypertension and hearing loss was not substantially altered by a variety of potential influencing factors, including study region (Europe, Asia, South America, Africa), age distribution (<40 years, 40–50 years, 50–60 years, >60 years), type of study (cross-sectional, case–control), proportion of women (<50, >50%), sample size (<10,000, >10,000), year of publication (2000–2009, 2010–2019, 2020–2023), and diagnostic criteria for hearing loss (>20 dB, ≥26 dB, other). At the same time, subgroup analyses were conducted according to whether confounding factors such as occupational exposure, diabetes mellitus, hyperlipidemia, and ototoxic drug use were excluded from the inclusion of the study population, and the same study was conducted in the study of exclusion of diabetes mellitus and hyperlipidemia, so subgroup analyses were conducted only once for these 2 confounding factors. Because there was only 1 study that excluded smoking and no study that excluded alcohol consumption, these two confounders were not subgroup analyzed. Despite our comprehensive subgroup analyses, the outcomes of the meta-regression exhibited *p*-values exceeding 0.05 for all subgroups, indicating our inability to pinpoint significant sources of inter-study heterogeneity.

The detailed outcomes of the subgroup analysis were presented in Table 3 and Annexure 8. Subgroup analysis across various study regions revealed that the effect measure for the European cohort did not achieve statistical significance at *p* > 0.05, while the effect measure for the African region surpassed that of other regions. Analysis of different age groups demonstrated that the effect indicator for the 50–60 years cohort did not reach statistical significance at *p* > 0.05, and the distinctions among the effect indicator for the other age groups were marginal.

In the subgroup analyses across various study types, the *p*-value for the effect indicator in the case–control studies exceeded 0.05, rendering the effect indicator statistically insignificant. When subgroup analysis was performed for different gender compositions and sample sizes, we found that the difference in effect values between groups was small, showing relatively consistent results. Subgroup analysis of year of publication showed that studies published during 2000–2009 had higher effect values than those published in later years. Subgroup analysis of the different hearing diagnostic criteria showed higher effect values in the >20 dB group than in the others. In the subgroup analysis of whether or not to include noise occupational exposure studies, the excluded group effect value was significantly higher than the unexcluded group. In the subgroup analysis excluding diabetes/hyperlipidemia, the excluded group effect value of *p* > 0.05 was not statistically significant, and the difference between the effect value of the non-excluded group and the total combined effect value was small. In the subgroup analysis excluding ototoxic drug use, the effect value of the non-excluded group was *p* > 0.05 and the effect value was not statistically significant, and the effect value of the excluded group was close to the total combined effect value.

## Discussion

The link between hypertension and hearing loss is still controversial, with some studies suggesting a significant correlation between hypertension and hearing loss. Some related mechanism research may also explain this correlation. Some studies suggest that hypertension may lead to hyperviscosity syndrome triggering hearing loss (37). At the same time, hypertension will accelerate the process of age-related hearing loss (38). It has also been shown that hypertension can affect auditory signaling by affecting the microcirculation of the vascular stria (39, 40). Animal studies have shown that a decrease in intracochlear potentials and hearing loss can be observed immediately after a hypoxic event (41). It has also been shown that hypertension can trigger hearing loss by altering the concentration of ions inside and outside the cell membrane (42). Nevertheless, these specific physiological mechanisms have not been thoroughly studied. This paper discusses the relationship between hypertension and hearing loss by means of meta-analysis, aiming at providing motivation for subsequent researchers and promoting them to carry out more extensive and in-depth mechanism research.

In this investigation, we probed the potential correlation between hypertension and auditory impairment. Employing meta-analysis, we unearthed a notably elevated likelihood of hypertension among individuals experiencing hearing loss [OR (95%CI): 1.849 (1.549, 2.208), *I*^2^ = 98%, *p* < 0.1]. Notably, subsequent to adjustments and amalgamation of effect sizes, while a connection between hypertension and hearing loss persisted, this relationship exhibited a relative attenuation. In the subgroup analyses the European group, the case–control study group, and the 50–60 years group were significantly lower than the combined effect values, and the African group, the >20 dB group, and the group controlling for noise exposure were significantly higher than the combined effect values. Due to the different influences adjusted for in the different studies and the lack of raw data, we were unable to determine which influences were responsible for this difference. The subgroup analyses of this meta-analysis showed that factors such as region, study method, diagnostic criteria, and noise exposure had a greater influence on the effect values. This suggests that future studies could control for these aspects with a view to more accurately responding to the relationship between hypertension and hearing loss. However, some of the subgroup analyses (European group, African group, 50–60 years group, >60 years group, Case–control group, 2000–2009 group, >20 dB group) were based on very limited sample sizes, and we have reservations about their clinical reliability, which will need to be validated by more high-quality, large-sample, multicenter clinical trials in the future.

In our study, the difference in effect values between different age groups was not significant, suggesting that the association between hypertension and hearing loss does not change significantly with age. However, this contradicts the findings of existing studies. A cohort study with a follow-up period of up to 7 years showed an association between hypertension and hearing loss among younger participants, but no such association was observed in the >65 age group (43). In the subgroup analyses, the >60 years group was based on a very limited sample size, which may be one of the major reasons for this discrepancy in results, and which needs to be verified in future high-quality, large-sample, multicenter clinical trials.

Similarly, in the subgroup analysis by year of publication, we also found higher effect values for studies published between 2000 and 2009. However, given the relatively small number of studies in this subgroup, we cannot rule out the possibility that this result may have been influenced by happenstance. Furthermore, the variances in research could stem from disparities in research methodologies across distinct epochs and advancements in medical technology. For example, with the improvement of medical level and the enhancement of people’s awareness of health care, more early and standardized treatment of hypertension will reduce the connection between hypertension and hearing loss. These external factors may also be an important reason for the difference in effect values between subgroups.

In the gender subgroup analysis, while we noted a reduced impact of female representation on the effect magnitudes, we encountered disparities when compared to findings from other investigations. Many studies have has highlighted that male gender represents an autonomous risk factor for auditory impairment, particularly within the context of hypertension-related hearing loss, with this correlation being more accentuated in males (44–46). However, within the current meta-analysis, owing to constraints within the raw data, we were unable to conduct a thorough examination of the correlation between hypertension and hearing loss specific to male and female participants. Instead, we relied on the proportion of female participants as an indicator for our analysis. This methodology may not accurately capture the true influence of gender in the association between hypertension and hearing loss, potentially resulting in incongruities with prior studies. To more precisely assess the role of gender in the link between hypertension and hearing loss, we propose the adoption of more intricate data analysis techniques in future research, allowing for separate exploration of the relationship between hypertension and hearing loss in both men and women.

Because the diagnostic criteria for hearing loss have been defined differently over time, there were differences in the diagnostic criteria among the included articles, but according to the latest diagnostic criteria of the WHO World Report on Hearing, the latest diagnostic criteria were used in this article: hearing loss was defined as a mean hearing threshold of ≥20 dB (14). The latest diagnostic criteria included those defined by previous diagnostic criteria (e.g., >20 dB, ≥26 dB), so the bias of the articles was not affected by this. The updated diagnostic criteria include those defined by previous diagnostic criteria (e.g., >20 dB, ≥26 dB) and therefore do not affect the bias of the article. However, due to the updated diagnostic criteria, some people with hearing loss may be missed. In the subgroup analysis, when >20 dB was used as the criterion for hearing loss, the effect value was significantly higher than that of the other groups. This finding suggests that there may be a stronger association between hypertension and mild hearing loss. This is consistent with the findings of Umesawa, which also showed a higher prevalence of mean mild hearing loss in hypertensive subjects compared with subjects without hypertension; however, no such correlation was found between hypertension and mean moderate to severe hearing loss. However, the >20 dB group reduces its clinical reliability based on the limited number of studies.

There was a significant high degree of heterogeneity among the studies included in this meta-analysis (*I*^2^ = 98%, *p* < 0.1), which adds to the difficulty of interpreting the results. The literature indicated that high heterogeneity can be identified and treated by sensitivity analysis, meta-regression analysis, and subgroup analysis (47). Through sensitivity analysis, we identified three studies, namely Aimoni, Kuang, and Wang, as potential sources of heterogeneity. After their exclusion, the remaining nine studies underwent heterogeneity testing, revealing an absence of detectable heterogeneity (*I*^2^ = 0%, *p* = 0.46). The three studies differed in terms of year of publication, region, type of study, diagnostic criteria, and sample size, with the common denominator being that the percentage of women was less than 50%. However, despite subgroup meta-analyses (including region, age, type of study, percentage of females, sample size, year of publication, diagnostic criteria for hearing, and exclusion of relevant confounders (occupational exposures, diabetes mellitus, ototoxic medication use, etc.) at the time of inclusion of study participants) and meta-regression analyses, we were unable to identify a definitive source of heterogeneity. The three studies differed in terms of year of publication, region, type of study, diagnostic criteria, and sample size, with the common denominator being that the percentage of women was less than 50%. It is worth noting that the level of heterogeneity does not directly determine the reliability of the results of the meta-analysis, but rather requires that we interpret the results with due consideration of the variability of the results and potential sources of heterogeneity (48).

A correlation between blood pressure variability and hearing loss has been observed in male patients, with systolic blood pressure levels positively correlating with mid-and high-frequency hearing loss, but no significant correlation was observed between diastolic blood pressure and changes in hearing (49). A case–control study suggests that elevated lipid levels may be a causative factor in hearing impairment and may influence the degree of hearing loss (50). The use of different hypertensive medications can also have an effect on hearing loss, and it has been suggested that telmisartan is associated with a reduction in certain types of hearing loss in hypertensive patients (51). A number of studies have also reported the association of diabetes, smoking, alcohol consumption, and ototoxic drug use with hearing loss (52–55). We believe that the above confounding factors may be the source of the high heterogeneity in this paper; unfortunately, we were unable to directly test these hypotheses due to insufficient disclosure of information. It is recommended that future studies should aim to conduct higher-standard, large-scale case–control studies with exhaustive elaboration of the relevant confounders of the association between hypertension and hearing loss, with a view to obtaining more precise and comprehensive findings.

Despite the significant and high degree of heterogeneity, our sensitivity analyses were successful in identifying and removing the major sources of heterogeneity, and the effect sizes of the studies did not change significantly after removing these studies. Therefore, in combination with these analyses, we believe that the conclusion of a positive association between hypertension and hearing loss remains reliable based on the current evidence base.

In addition to high heterogeneity, this study has the following limitations: (1) This paper conducted subgroup analyses based on whether or not noise exposure, diabetes, and other confounders were excluded at the time of inclusion of the study subjects, but due to the unavailability of raw data, the studies varied in terms of the extent to which they controlled for confounders, and only some of them were adjusted for OR, and the adjustment for the factors varied from one study to the next, so this paper was unable to conduct a more detailed and precise analysis. (2) Some of the subgroup analyses included small sample sizes and require careful consideration of their clinical reliability. (3) The design of the current included studies was cross-sectional or case–control, and because of their inherent design characteristics, they therefore had limitations in exploring associations between variables, especially the inability to accurately determine temporal order and causality (56). It is recommended that future prospective cohort studies should directly investigate the temporal relationship between hypertension and hearing loss. (4) Although we did not detect publication bias through funnel plots, Egger and Begg tests, there may be undetected publication bias due to the limitations of the design of the studies included in this article.

## Conclusion

In conclusion, our study initially revealed a significant association between hypertension and hearing loss. From a clinical perspective, the correlation between hypertension and hearing impairment not only reveals the potential harm of hypertension on the auditory system, but also provides a new perspective on the health management of hypertensive patients. When diagnosing and treating hypertensive patients, doctors need to pay attention to their hearing status in addition to their cardiovascular health. Through regular hearing screening, hypertension-related hearing loss can be detected and intervened in a timely manner, thus improving patients’ quality of life.

## Data Availability

The original contributions presented in the study are included in the article/Supplementary material, further inquiries can be directed to the corresponding author.
