# Supplementary material for: Association between hypertension and hearing loss: a systemic review and meta-analysis
Source: Front Neurol. 2025 Jan 7;15:1470997. doi: 10.3389/fneur.2024.1470997 (PMC11748549; doi:10.3389/fneur.2024.1470997)
Supplement: Supplementary file 1 [file Supplementary_file_1.docx]

**Supplementary Information**

**Annexure 1: Inclusion and exclusion criteria Research**

**Question: Association between hypertension and hearing loss**

|  | Inclusion | Exclusion |
| --- | --- | --- |
| Participants | The average hearing threshold ≥ 20dB  Systolic blood pressure (SBP)≥ 140 mmHg or diastolic blood pressure (DBP)≥ 90 mmHg or reported the use of antihypertensive drugs  All gender  All age groups | Diagnostic criteria are not clear |
| Disease | Hearing loss  Hypertension | Suffering from other diseases that may cause hearing loss. |
| Outcome | OR and 95% CIs or relevant data to calculate OR and 95%CIs | Incomplete research data |
|  | Sub-group analysis according to age, gender, region, publication year, etc. |  |
| Study Designs | Case-control studies, cross-sectional studies, cohort studies | Qualitative, policy, opinion, case-reports, review, case studies |
|  | Geography-Global level  Date of Search- Publish till March 23, 2024  English Language  Human studies | Animal experiment |

**Annexure 2a：Quality assessment of included case-control with the use of NOS quality assessment tool**

|  | **Selection** | | | | **Comparability** | **Exposure** | | |  |
| --- | --- | --- | --- | --- | --- | --- | --- | --- | --- |
|  | Q1 | Q2 | Q3 | Q4 | Q1 | Q1 | Q2 | Q2 |  |
| Aimoni 2010 | * | * |  |  | ** | * | * |  | ******* |
| Moraes 2006 | * | * | * |  | ** | * | * |  | ******** |

**Selection**

Q1 Is the case definition adequate?

a) yes, with independent validation*

b) yes, eg record linkage or based on self reports

c) no description

Q2 Representativeness of the cases

a) consecutive or obviously representative series of cases*

b) potential for selection biases or not stated

Q3 Selection of Controls

a) community controls*

b) hospital controls

c) no description

Q4 Definition of Controls

a) no history of disease (endpoint) *

b) no description of source

**Comparability**

Q1 Comparability of cases and controls on the basis of the design or analysis

a) study controls for _______________ (Select the most important factor.) *

b) study controls for any additional factor *

(This criteria could be modified to indicate specific control for a second important factor.)

**Exposure**

Q1 Ascertainment of exposure

a) secure record (eg: surgical records )*

b) structured interview where blind to case/control status*

c) interview not blinded to case/control status

d) written self report or medical record only

e) no description

Q2 Same method of ascertainment for cases and controls

a) yes *

b) no

Q3 Non-Response rate

a) same rate for both groups *

b) non respondents described

c) rate different and no designation

**Annexure 2b: Quality assessment of included cross-sectional with the use of AHRQ quality assessment tool**

| **Author (Year)** | **Q1** | **Q2** | **Q3** | **Q4** | **Q5** | **Q6** | **Q7** | **Q8** | **Q9** | **Q10** | **Q11** | **scores** |
| --- | --- | --- | --- | --- | --- | --- | --- | --- | --- | --- | --- | --- |
| Samelli 2021 | Y | Y | Y | Y | Y | N | Y | Y | UN | Y | N | 8 |
| Wang 2018 | Y | Y | Y | Y | Y | Y | Y | Y | UN | N | N | 8 |
| Kuang 2019 | Y | Y | Y | Y | Y | Y | Y | Y | UN | Y | N | 9 |
| Zhou 2019 | Y | Y | Y | Y | Y | Y | Y | Y | UN | Y | N | 9 |
| Ramatsoma 2022 | Y | Y | Y | N | Y | N | Y | Y | UN | N | N | 6 |
| Zhang 2023 | Y | Y | Y | Y | Y | Y | Y | Y | UN | Y | N | 9 |
| Oh 2014 | Y | Y | Y | Y | Y | Y | Y | Y | UN | N | N | 8 |
| Guo 2021 | Y | Y | Y | Y | Y | N | Y | Y | UN | N | N | 7 |
| Hara 2020 | Y | Y | Y | N | Y | Y | Y | Y | UN | Y | N | 8 |
| Umesawa 2019 | Y | Y | Y | Y | Y | Y | Y | Y | UN | Y | N | 9 |

Y: Yes; N: NO; UN: Unclear

Q1 Define the source of information (survey, record review)

Q2 List inclusion and exclusion criteria for exposed and unexposed subjects (cases and controls) or refer to previous publications

Q3 Indicate time period used for identifying patients

Q4 Indicate whether or not subjects were consecutive if not population-based

Q5 Indicate if evaluators of subjective components of study were masked to other aspects of the status of the participants

Q6 Describe any assessments undertaken for quality assurance purposes (e.g., test/retest of primary outcome measurements)

Q7 Explain any patient exclusions from analysis

Q8 Describe how confounding was assessed and/or controlled.

Q9 If applicable, explain how missing data were handled in the analysis

Q10 Summarize patient response rates and completeness of data collection

Q11 Clarify what follow-up, if any, was expected and the percentage of patients for which incomplete data or follow-up was obtained

**Annexure 3：The adjusted search terms as per searched electronic databases [as of 23.03.2024]**

| **Database** | **No** | **Search Query** | **Results** |
| --- | --- | --- | --- |
| **PubMed/MEDLINE** | | | |
|  | #1 | (((((hearing loss[MeSH Terms]) OR (hearing loss[Title/Abstract])) OR (Hypoacusis[Title/Abstract])) OR (Hearing Impairment*[Title/Abstract])) OR (Deafness*[Title/Abstract])) OR (hearing disability[Title/Abstract]) | [114,704](https://pubmed.ncbi.nlm.nih.gov/?term=(((((hearing+loss%5bMeSH+Terms%5d)+OR+(hearing+loss%5bTitle/Abstract%5d))+OR+(Hypoacusis%5bTitle/Abstract%5d))+OR+(Hearing+Impairment*%5bTitle/Abstract%5d))+OR+(Deafness*%5bTitle/Abstract%5d))+OR+(hearing+disability%5bTitle/Abstract%5d)&sort=&filter=dates.1000/1/1-2024/3/22&size=100) |
|  | #2 | ((((hypertension[MeSH Terms]) OR (hypertension[Title/Abstract])) OR (high blood pressure*[Title/Abstract])) OR (hypertensive[Title/Abstract])) OR (blood pressure[Title/Abstract]) | [799,019](https://pubmed.ncbi.nlm.nih.gov/?term=((((hypertension%5bMeSH+Terms%5d)+OR+(hypertension%5bTitle/Abstract%5d))+OR+(high+blood+pressure*%5bTitle/Abstract%5d))+OR+(hypertensive%5bTitle/Abstract%5d))+OR+(blood+pressure%5bTitle/Abstract%5d)&sort=&filter=dates.1000/1/1-2024/3/22&size=100) |
|  | #3 | #1 AND #2 | 1712 |
| **Embase** | | | |
|  | #1 | 'arterial hypertension':ab,ti OR 'blood pressure, high':ab,ti OR 'cardiovascular hypertension':ab,ti OR 'high blood pressure':ab,ti OR 'hypertensive disease':ab,ti OR 'systemic hypertension':ab,ti OR 'hypertension':ab,ti | [756,345](https://ras.cdutcm.edu.cn:7080/s/com/embase/www/G.https/) |
|  | #2 | 'auditory defect':ab,ti OR 'deaf':ab,ti OR 'deafness':ab,ti OR 'hard of hearing':ab,ti OR 'hearing damage':ab,ti OR 'hearing defect':ab,ti OR 'hearing difficulty':ab,ti OR 'hearing loss':ab,ti OR 'hypacousia':ab,ti OR 'hypacousis':ab,ti OR 'hypacusia':ab,ti OR 'hypacusis':ab,ti OR 'hypakousia':ab,ti OR 'hypakusis':ab,ti OR 'hypoacousia':ab,ti OR 'hypoacousis':ab,ti OR 'hypoacusia':ab,ti OR 'hypoacusis':ab,ti OR 'hypoakusis':ab,ti OR 'impaired hearing':ab,ti OR 'hearing impairment':ab,ti | [112,157](https://ras.cdutcm.edu.cn:7080/s/com/embase/www/G.https/) |
|  | #3 | #1 AND #2 | 1,877 |
| **Web of Science** | | | |
|  | #1 | (hypertension OR high blood pressure* OR hypertensive OR blood pressure ) (All Fields) | 454,797 |
|  | #2 | (hearing loss OR hearing loss OR Hypoacusis OR Hearing Impairment* OR Deafness* OR hearing disability ) (All Fields) | [63,964](https://ras.cdutcm.edu.cn:7080/s/cn/clarivate/webofscience/G.https/wos/woscc/summary/aae7d097-1673-4c1d-a0ce-a36d561e64e5-eb401c59/relevance/1) |
|  | #3 | #1 AND #2 | 1131 |
| **Scopus** | | | |
|  | #1 | ( TITLE-ABS-KEY ( "hearing loss" ) OR TITLE-ABS-KEY ( hypoacusis ) OR TITLE-ABS-KEY ( "Hearing Impairment*" ) OR TITLE-ABS-KEY ( deafness* ) OR TITLE-ABS-KEY ( "hearing disability" ) ) | 163,044 |
|  | #2 | ( TITLE-ABS-KEY ( hypertension ) OR TITLE-ABS-KEY ( "high blood pressure*" ) OR TITLE-ABS-KEY ( hypertensive ) OR TITLE-ABS-KEY ( blood AND pressure ) ) | 1,469,956 |
|  | #3 | #1 AND #2 | 5967 |

**Annexure 4: List of excluded reviews**

| NO | Title | Reason for exclusion |
| --- | --- | --- |
| 1 | Associations between cardiovascular disease and its risk factors with hearing loss-A cross-sectional analysis | No data available |
| 2 | Cardiovascular and Thromboembolic Risk Factors in Idiopathic Sudden Sensorineural Hearing Loss: A Case-Control Study | Different interventions or outcomes |
| 3 | Associations Between Cardiovascular Risk Factors and Audiometric Hearing: Findings From the Canadian Longitudinal Study on Aging | Different interventions or outcomes |
| 4 | A study on early hearing impairment with essential hypertension | No data available |
| 5 | Cardiovascular risk factors and hearing loss: The HUNT study | Different interventions or outcomes |
| 6 | Cardiovascular Risk Factors and Sudden Sensorineural Hearing Loss: A Case-Control Study | No data available |
| 7 | Idiopathic sudden sensorineural hearing loss: evolution in the presence of hypertension, diabetes mellitus and dyslipidemias | No data available |
| 8 | Longitudinal Blood Pressure Patterns From Mid- to Late Life and Late-Life Hearing Loss in the Atherosclerosis Risk in Communities Study | Different interventions or outcomes |
| 9 | Prevalence and risk factors of sensorineural hearing loss in patients with systemic hypertension | Different interventions or outcomes |
| 10 | Recovery From Idiopathic Sudden Sensorineural Hearing Loss: Association With Cardiovascular Disease Risk | Study Design not adequate |
| 11 | Idiopathic sudden sensorineural hearing loss: cardiovascular risk factors do not influence hearing threshold recovery | Study Design not adequate |
| 12 | Age-related hearing loss in the Korea National Health and Nutrition Examination Survey | No data available |
| 13 | Characteristics and prognosis analysis of bilateral sudden sensorineural hearing loss: A retrospective case–control study | Study Design not adequate |
| 14 | The Relation of Hearing in the Elderly to the Presence of Cardiovascular Disease and Cardiovascular Risk Factors | No data available |
| 15 | Assessment of association between cardiovascular disease and its risk factors with hearing loss | No data available |
| 16 | Low-Frequency Audiometric Notch and Vascular Risk in Age-Related Hearing Loss | Study Design not adequate |
| 17 | Association of Cardiovascular Comorbidities With Hearing Loss in the Older Old | Study Design not adequate |
| 18 | Chronic Noise Exposure, High-Frequency Hearing Loss, and Hypertension among Automotive Assembly Workers | Study Design not adequate |
| 19 | Evaluation of heart rate variability and night-time blood pressure measurements in patients with idiopathic sudden sensory nervous hearing loss | Study Design not adequate |
| 20 | Probable Association of Hearing Loss, Hypertension and Diabetes Mellitus in the Elderly | No data available |
| 21 | Hypertension, Diuretic Use, and Risk of Hearing Loss | Study Design not adequate |
| 22 | A Prospective Study of Cardiovascular Risk Factors and Incident Hearing Loss in Men | No data available |
| 23 | High-frequency hearing loss, occupational noise exposure and hypertension: a cross-sectional study in male workers | No data available |
| 24 | Effects of Hypertension on Hearing | No data available |
| 25 | Hypertension is associated with dysfunction of both peripheral and central auditory system | Study Design not adequate |
| 26 | Positive Associations between Bilateral High-Frequency Hearing Loss and Hypertension Risk in Short-Term Occupational Noise Exposure | No data available |
| 27 | A study of noise-induced hearing loss and blood pressure in steel mill workers | Study Design not adequate |
| 28 | Angiotensin-converting enzyme gene polymorphisms and hypertension in occupational noise exposure in Egypt | Study Design not adequate |
| 29 | ELSA-Brasil: a 4-year incidence of hearing loss in adults with and without hypertension | No data available |
| 30 | Sensorineural Hearing Loss among Hypertensives | No data available |
| 31 | Contributing factors to high prevalence of hearing impairment in the Elias Motsoaledi Local Municipal area, South Africa: A rural perspective | No data available |
| 32 | Association between high systolic blood pressure and objective hearing impairment among Japanese adults: a facility-based retrospective cohort study | No data available |
| 33 | Hearing Problems in Indonesia: Attention to Hypertensive Adults | Do not meet the diagnostic criteria |
| 34 | Hypertension and hearing impairment in workers of iron and steel industry | Do not meet the diagnostic criteria |
| 35 | Prevalence of hearing impairment and its correlates among a group of hospitalized chronically ill elderly patients in Alexandria, Egypt | Do not meet the diagnostic criteria |
| 36 | Association of Midlife Hypertension with Late-Life Hearing Loss | Do not meet the diagnostic criteria |
| 37 | Etiological analysis of patients with sudden sensorineural hearing loss: a prospective case–control study | Study Design not adequate |
| 38 | Association of hypertension and age-related sensorineural hearing loss among elderly: A cross-sectional study | Do not meet the diagnostic criteria |
| 39 | Relationship of cardiovascular disease risk and hearing loss in a clinical population | Study Design not adequate |
| 40 | Hearing loss and hypertension: exploring the linkage | No data available |
| 41 | Hypertension associated with hearing health problems among Canadian adults aged 19 to 79 years | No data available |
| 42 | Age-related hearing loss and blood pressure | Study Design not adequate |
| 43 | A case-control study of occupational noise exposure induced high-frequency hearing loss and the risk of hypertension | No data available |

**Annexure 5：Forest map after removing high heterogeneity articles**

**
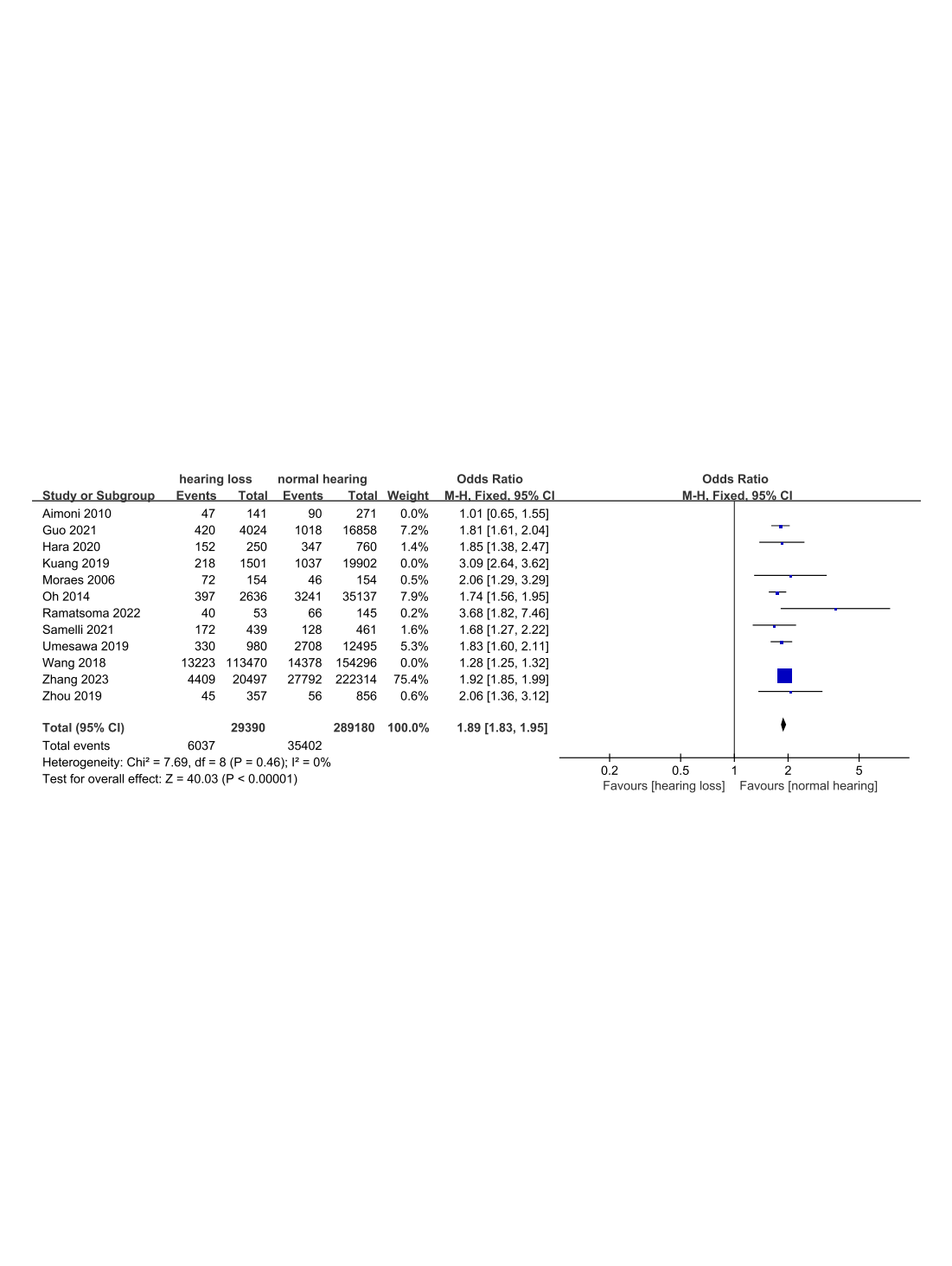
**

**Annexure6: Test results of begg bias of funnel diagram**

| Std_Eff | Coefficient | Std. err. | t | P>\|t\| | [95% conf. interval] | |
| --- | --- | --- | --- | --- | --- | --- |
| Slope bias | .3402288 | .0787981 | 4.32 | 0.002 | .1646556 | .515802 |
|  | 3.269887 | 2.306462 | 1.42 | 0.187 | -1.869231 | 8.409006 |

**Annexure7: Egger bias test results**

| Std_Eff | Coefficient | Std. err. | P>\|t\| | [95% conf. interval] | |
| --- | --- | --- | --- | --- | --- |
| Slope bias | 1.415188 | .1324065 | ＜0.001 | 1.120168 | 1.710208 |
|  | 5.953849 | 3.875607 | 0.155 | -2.681541 | 14.58924 |

**Annexure8: Sub-group analysis forest map**


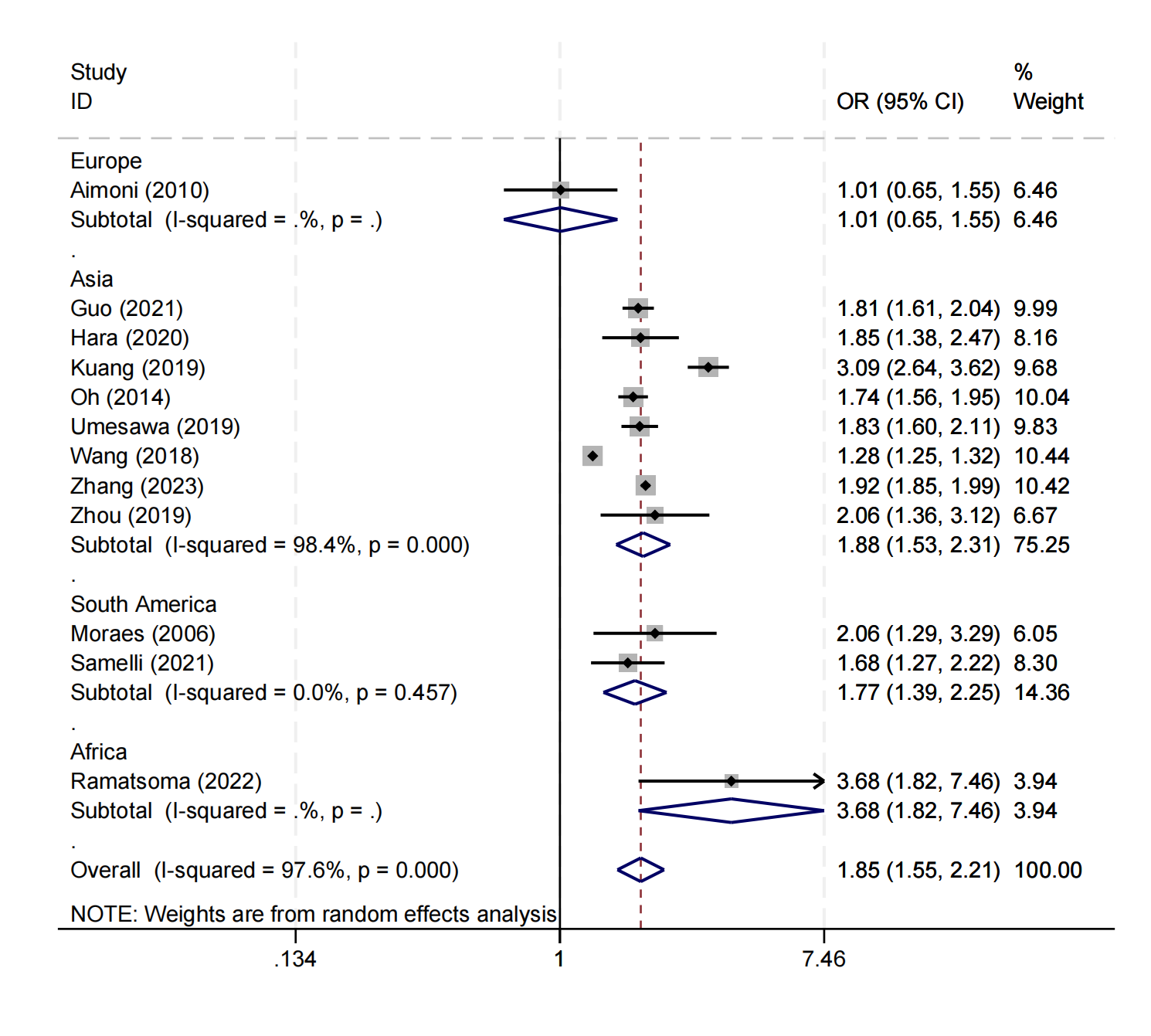


1. Sub-group analysis forest map-Country


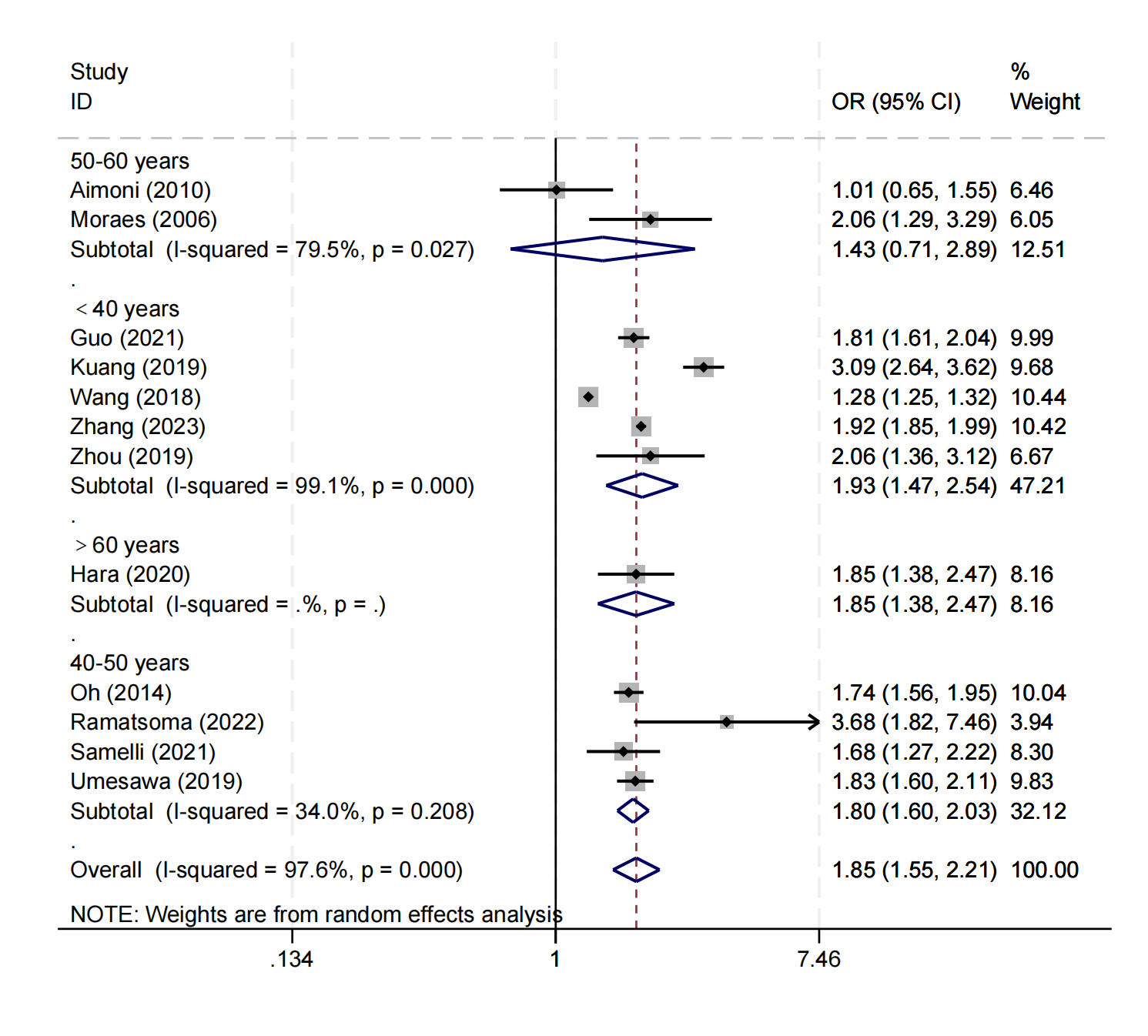


1. Sub-group analysis forest map-Age


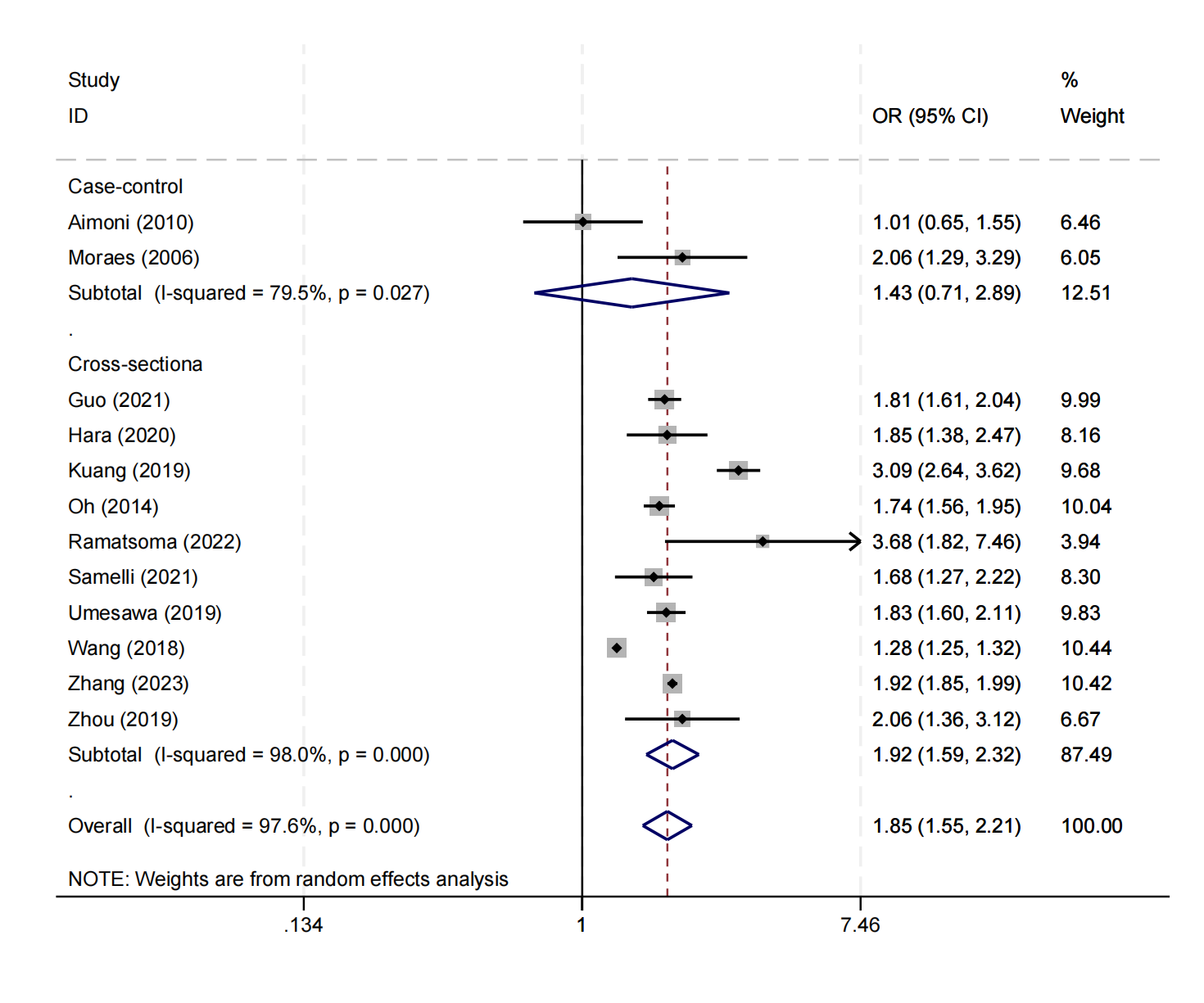


1. Sub-group analysis forest map-Research type


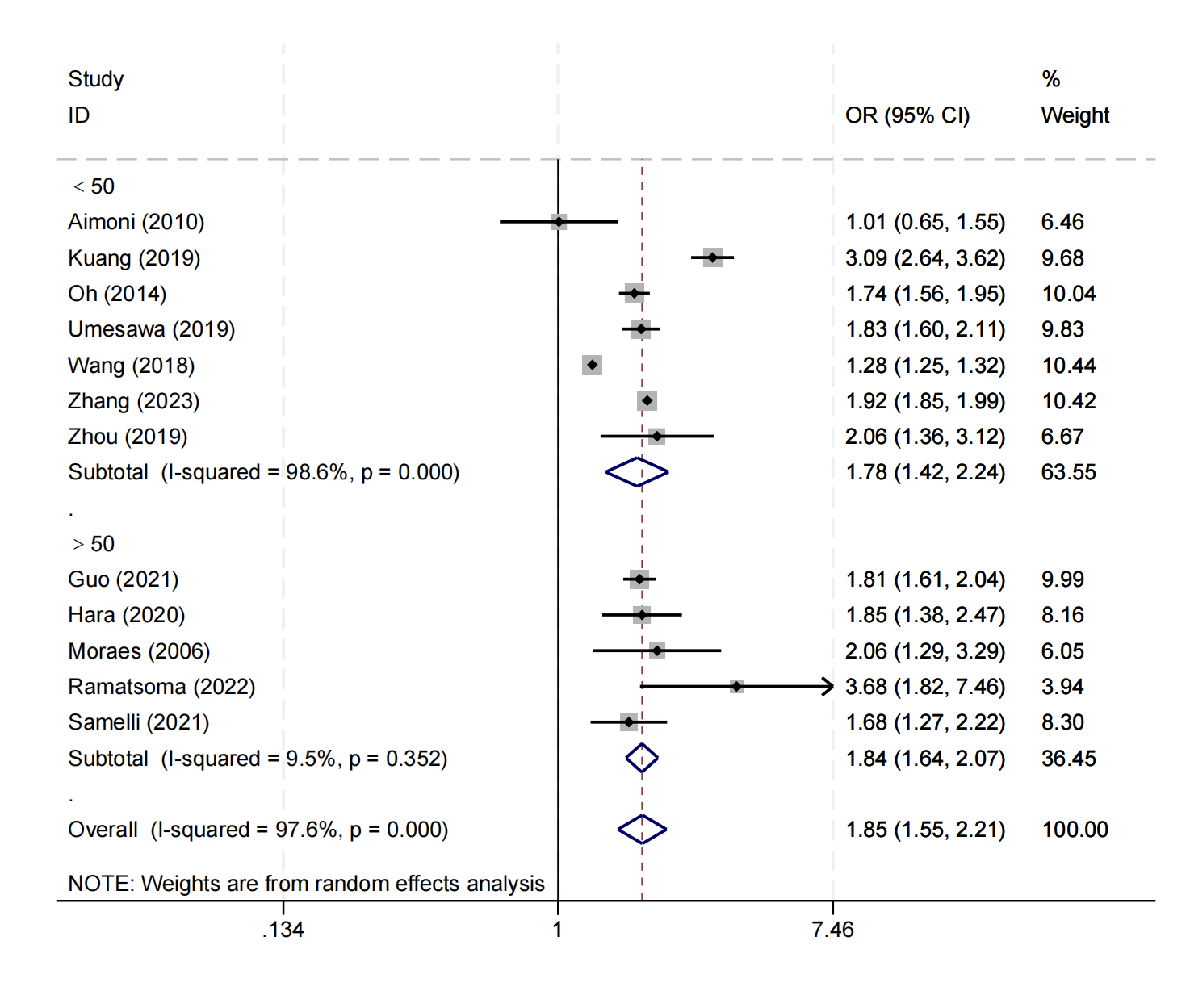


1. Sub-group analysis forest map-Gender (female proportion)


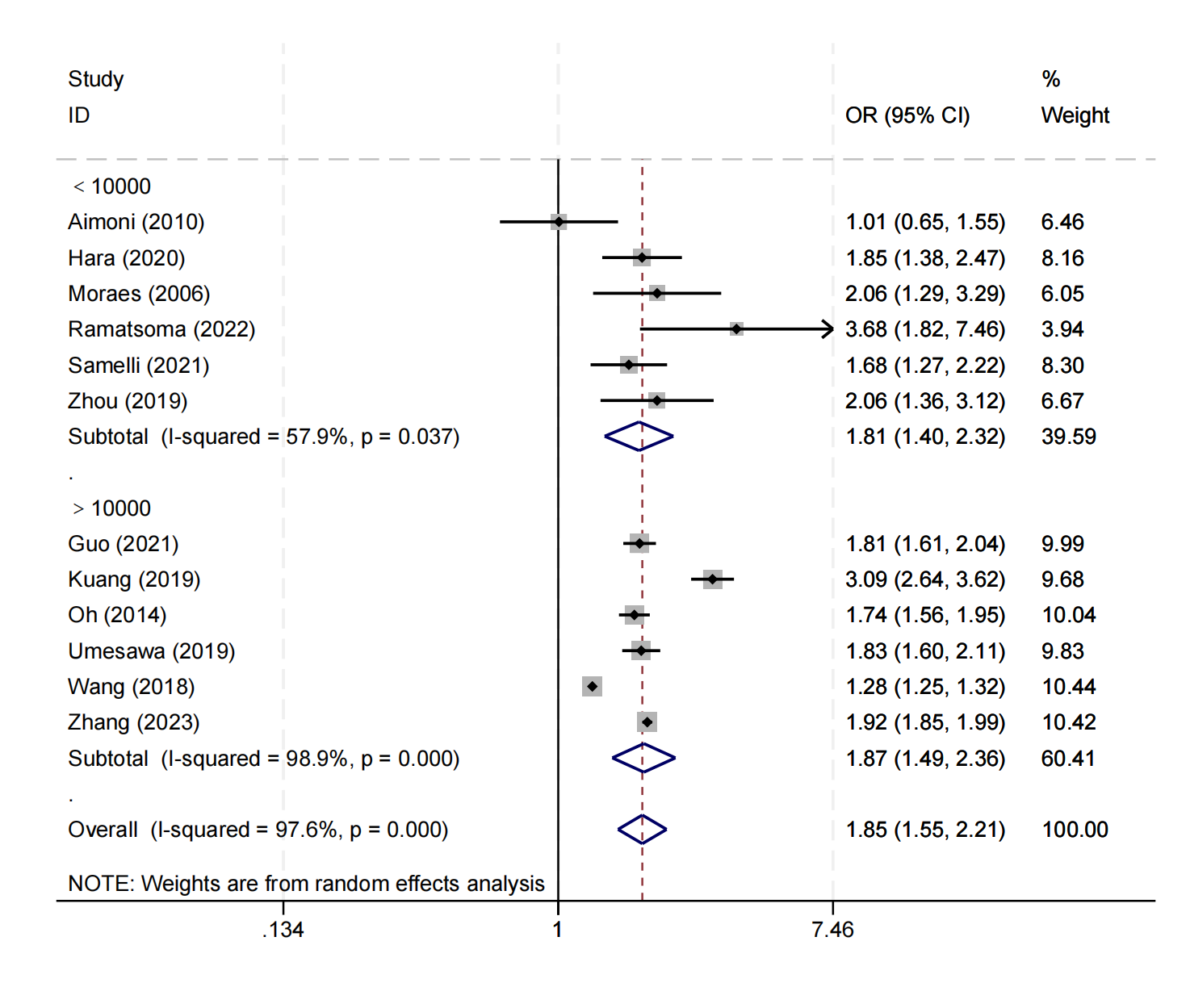


1. Sub-group analysis forest map-Sample size


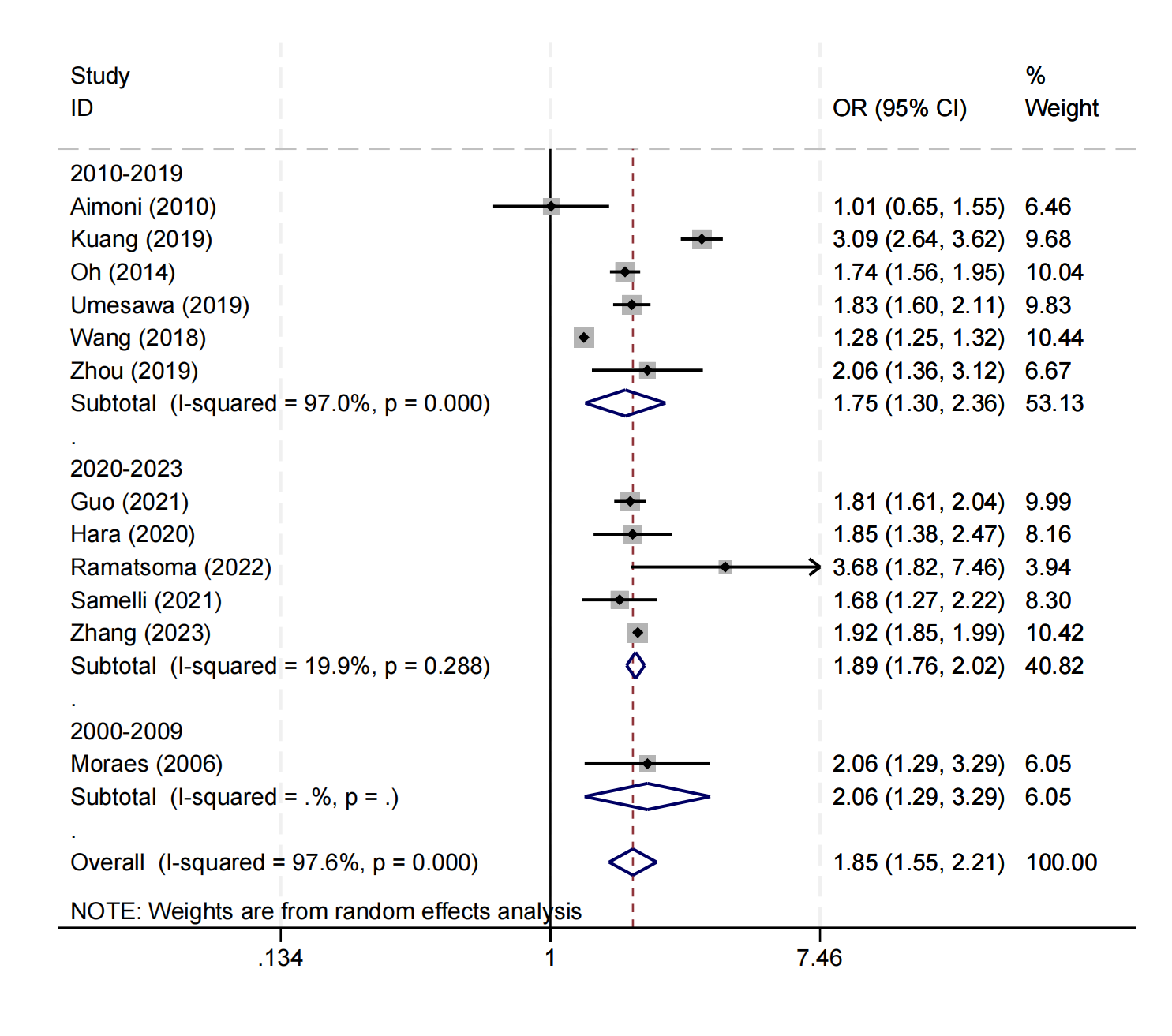


1. Sub-group analysis forest map-Publication year


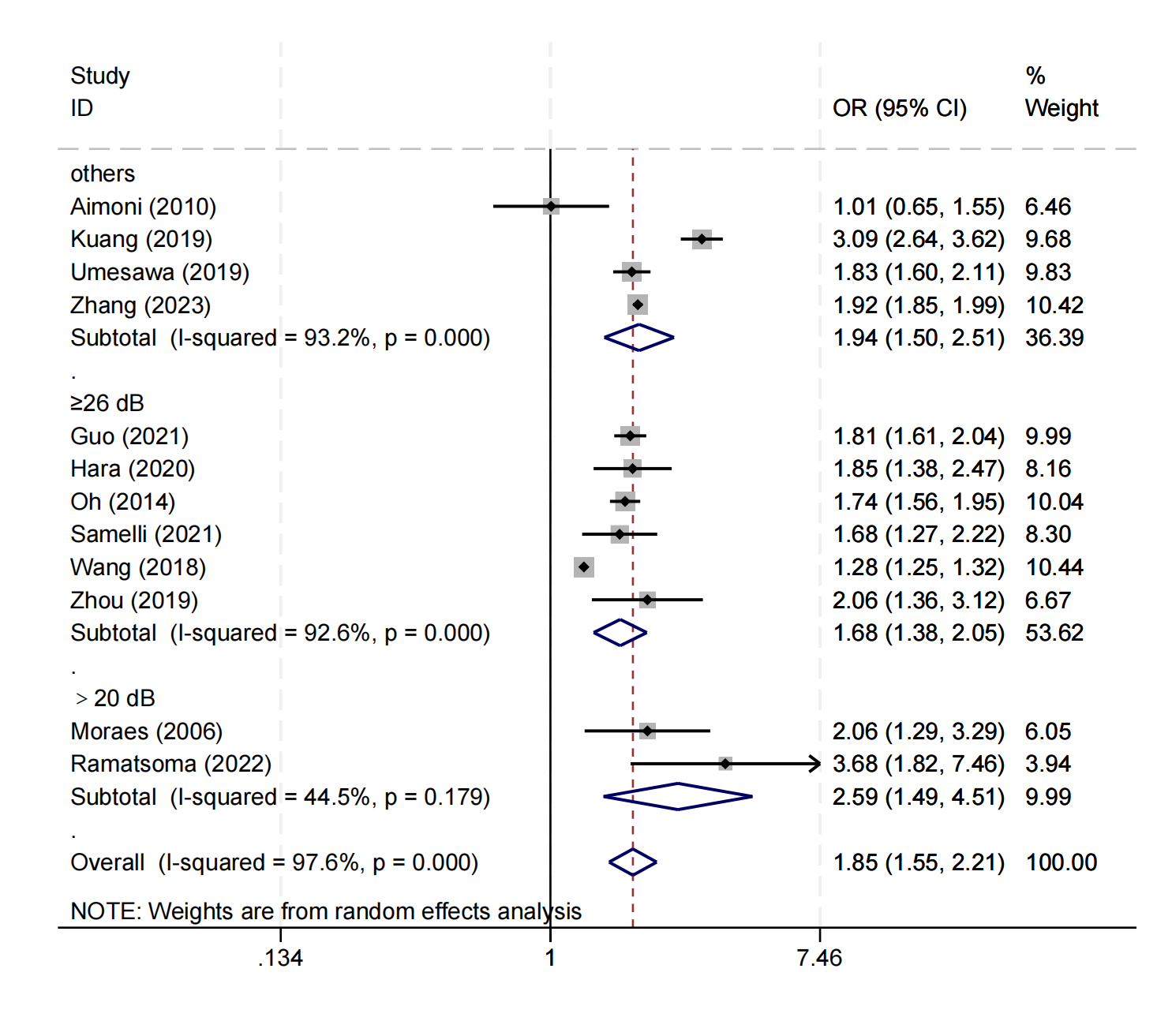


1. Sub-group analysis forest map-Diagnostic criteria of hearing


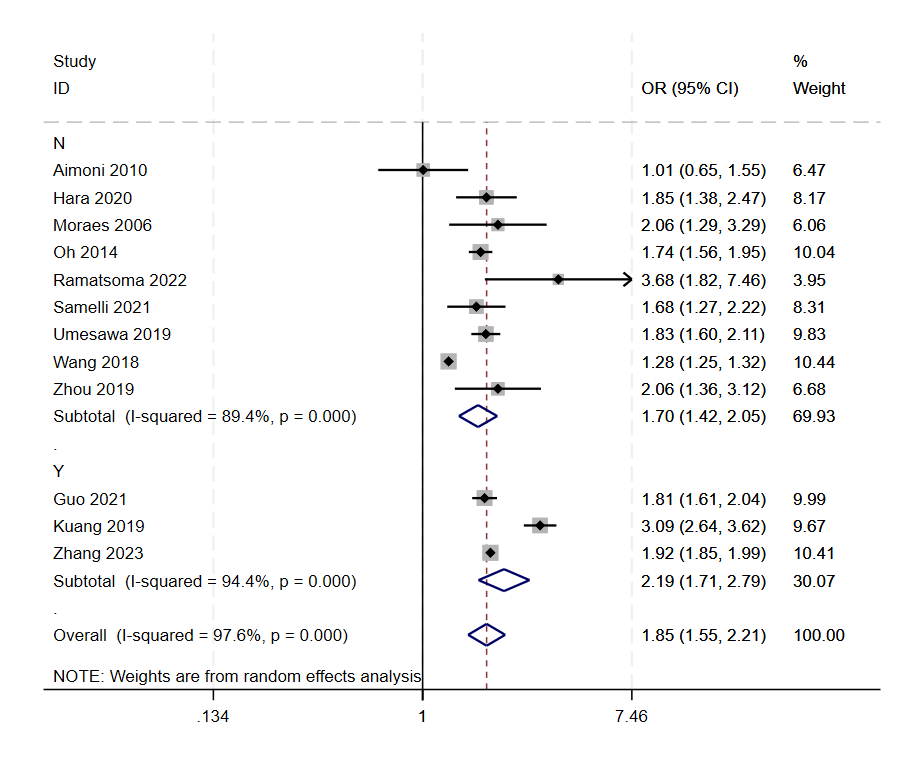


1. Sub-group analysis forest map- Exclusion of confounding factors when included in the study (occupational exposure). Y=Yes. N=No.


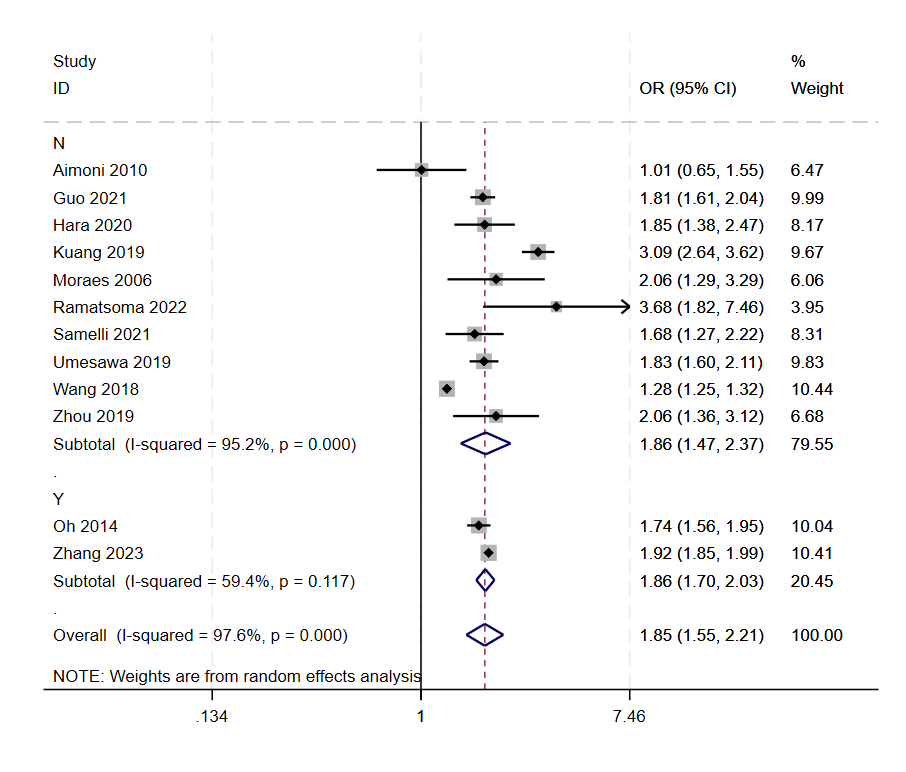


1. Sub-group analysis forest map- Exclusion of confounding factors when included in the study (diabetes/ hyperlipoidemia). Y=Yes. N=No.


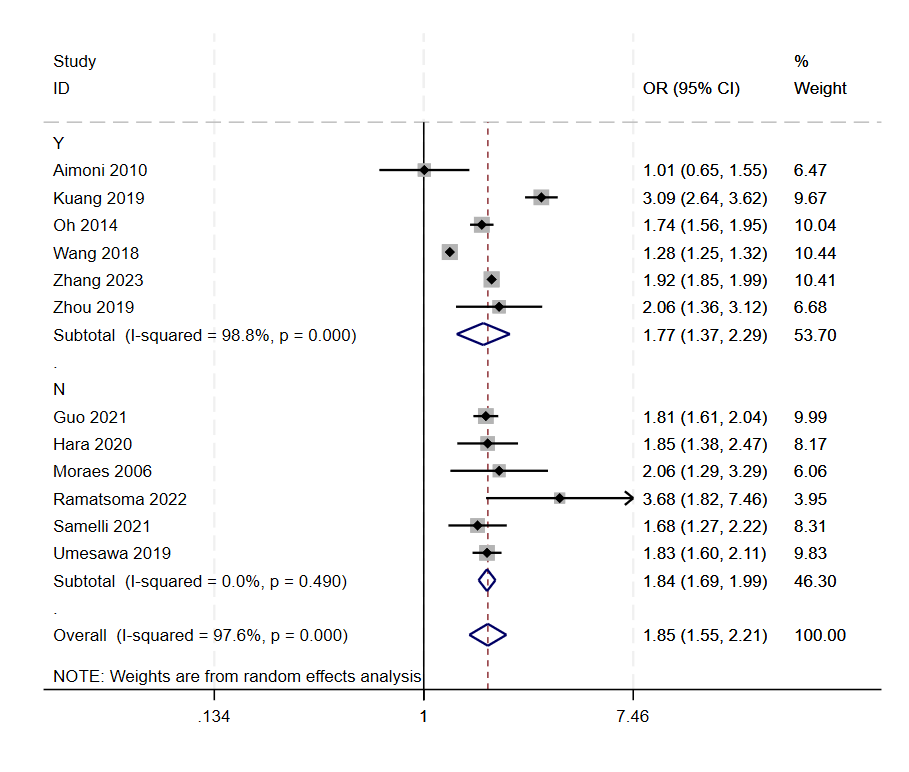


1. Sub-group analysis forest map- Exclusion of confounding factors when included in the study (ototoxicity drug use). Y=Yes. N=No.
